# Fibrillarin methylates H2A in RNA polymerase I trans-active promoters in *Brassica oleracea*

**DOI:** 10.3389/fpls.2015.00976

**Published:** 2015-11-06

**Authors:** Lloyd Loza-Muller, Ulises Rodríguez-Corona, Margarita Sobol, Luis C. Rodríguez-Zapata, Pavel Hozak, Enrique Castano

**Affiliations:** ^1^Unidad de Bioquímica y Biología Molecular de Plantas, Centro de Investigación Científica de YucatánMérida, Mexico; ^2^Department of Biology of the Cell Nucleus, Institute of Molecular Genetics of the Academy of Sciences of the Czech Republic, v.v.i.Prague, Czech Republic; ^3^Unidad de Biotecnología, Centro de Investigación Científica de YucatánMérida, Mexico

**Keywords:** histones, methylation, RNA polymerase I, *Brassica*, phosphoinositide

## Abstract

Fibrillarin is a well conserved methyltransferase involved in several if not all of the more than 100 methylations sites in rRNA which are essential for proper ribosome function. It is mainly localized in the nucleoli and Cajal bodies inside the cell nucleus where it exerts most of its functions. In plants, fibrillarin binds directly the guide RNA together with Nop56, Nop58, and 15.5ka proteins to form a snoRNP complex that selects the sites to be methylated in pre-processing of ribosomal RNA. Recently, the yeast counterpart NOP1 was found to methylate histone H2A in the nucleolar regions. Here we show that plant fibrillarin can also methylate histone H2A. In Brassica floral meristem cells the methylated histone H2A is mainly localized in the nucleolus but unlike yeast or human cells it also localize in the periphery of the nucleus. In specialized transport cells the pattern is altered and it exhibits a more diffuse staining in the nucleus for methylated histone H2A as well as for fibrillarin. Here we also show that plant fibrillarin is capable of interacting with H2A and carry out its methylation in the rDNA promoter.

## Introduction

The nucleolus is the largest structure inside the cell nucleus. The main function of this structure is ribosome biogenesis. This process involves transcription of rDNA, processing of rRNA and assembly of ribosomal proteins (Kressler et al., 1999). Ribosomal genes (rDNA) in eukaryotes are in a tandem arrayed of 100–1000s (depends on the species) copies at chromosomal loci, known as nucleolus organizer regions. Each rRNA gene is transcribed within the nucleolus by RNA polymerase I to produce a primary transcript that is processed to form the 18S, 5.8S, and 25–28S rRNAs (Nemeth and Langst, 2011). However, the nucleolus is also involved in several other processes like genetic silencing, cell cycle progression, senescence and biogenesis of snRNA and tRNAs (Jacobson and Pederson, 1998; Cockell and Gasser, 1999; Garcia and Pillus, 1999). In plants the nucleolus consists of four components: FCs, DFC, GC and the nucleolar vacuole (NV). Fibrillarin is a methyltransferase involved in the processing of the primary ribosomal transcript and is mainly located in the FC and DFC region of the nucleoli where it is directly involved in several steps of ribosome biogenesis (Rodriguez-Corona et al., 2015). Fibrillarin is known to be part of the snoRNP that methylate rRNA (Tollervey et al., 1993). Biochemical evidence for the process with eukaryotic fibrillarin is lacking but it has been demonstrated using aFib in order to recapitulate the methylation process on rRNA (Tran et al., 2003). High resolution crystal structure data from this complex has been obtained by several laboratories (Aittaleb et al., 2003; Oruganti et al., 2007; Ye et al., 2009) and have shown a well conserved overall structure (Rodriguez-Corona et al., 2015). The snoRNA acts like a guide to help direct aFib together with Nop56/58 and L7Ae that interact with the rRNA in order to methylate at specific sites. In eukariotes fibrillarin has been shown to form a complex with Nop56, Nop58, protein 15.5Ka and different guide RNAs like U3, U6, etc. The guide RNA recognizes specific regions to be methylated on rRNA. Fibrillarin is also involve in the earliest steps of ribosomal transcription initiation and this steps require the interaction with PI4,5P2 (Sobol et al., 2013; Yildirim et al., 2013) linking the rRNA processing with rRNA transcription initiation where PLC can inhibit transcription initiation (Yildirim et al., 2013). Overproduction of fibrillarin in mammalian cells can lead to alteration on ribosomal methylation and as a result there is an alteration in the process of translation. Highly methylated ribosomes surpass IRES leading to misread translation that results in some types of cancers (Marcel et al., 2013). In plants, fibrillarin has been shown to be part of the mediator of RNA polymerase II transcription (subunit 36a) (Backstrom et al., 2007). Two different RNA binding sites have been determined in fibrillarin from *Arabidopsis thaliana* (Rakitina et al., 2011). Plant fibrillarin has also been a link between both rRNA gene binding and pre-rRNA processing by analyzing the fractions containing the snoRNP complex in both promoter complex and rRNA cleavage sites (Saez-Vasquez et al., 2004). Moreover, plant umbravirus life cycle suggest the requirement of fibrillarin. Fibrillarin is redistributed upon infection to the cytoplasm and participates in the formation of viral ribonucleoproteins able to move through the plant phloem resulting in complete infection of the plant (Kim et al., 2007). Recently, fibrillarin has been shown to be involved in epigenetic nucleolar mechanism. Fibrillarin methylate histone H2A in yeast and human cells at position Q105 and this methylation is unique to the nucleolus (Tessarz et al., 2014). The FACT (facilitates chromatin transcription) is a protein complex known to facilitate transcription elongation of RNA pol II derived transcription where it has a preferential interaction to histone H2A/H2B dimers. In RNA pol I transcription FACT interacts preferentially with the methylated H2A to reorganized nucleosomes in the active promoters for rRNA (Tessarz et al., 2014). Nevertheless, the ribosomal promoter has been shown to differ significantly between mammalian and plants (Perry, 2005; Knight et al., 2014). We show that plant fibrillarin is also capable to methylate histone H2A while bound to the rDNA. Our results also showed that *in vivo* methylated histone H2A in *B. oleracea* can also be found at other locations besides the nucleolar regions, this modification in plants may have additional epigenetic roles than what is found in animal cells.

## Materials and Methods

### Maintenance and Propagation of Cell Culture

U2OS osteosarcoma cells were kept in DMEM with 10% fetal calf serum in 5% CO_2_/air, 37°C, humidified atmosphere.

### Antibodies

Rabbit polyclonal anti-H2A (Q105Met) was a kind gift from Tessarz et al. (2014). Rabbit Fibrillarin Antibody (H-140): Santa cruz sc-25397); Anti-Histone H2A antibody ChIP Grade (ab15653) Abcam. Anti-Histone H3 (mono methyl K4) antibody – ChIP Grade (ab8895). Goat Anti-Rabbit IgG H&L (Alexa Fluor 647) (ab150079) Abcam. (Goat anti-Rabbit IgG (H+L) Secondary Antibody, Alexa Fluor 488 conjugate (Invitrogen) (A-11008).

### Nucleotide Sequence Data base

Fibrillarin nucleotide sequence from *B. oleracea* (BoFib) was obtained from the database for *B. oleracea* (http://www.ocri-genomics.org/bolbase/) with the accession number: Bol39546. All other nucleotide sequence were obtained from NCBI: Saccharomyces cerevisiae fibrillarin (Nop1: CAA98572.1), Homo sapiens fibrillarin (HsFib: CAA39935.1) and *Arabidopsis thaliana* fibrillarins 1 and 2 (AtFib1: NP_568772.3, AtFib2: NP_567724.1; respectively).

### Plasmids

pET15b::Fibrillarin contain the sequence from *A. thaliana fibrillarin 2* (NP_567724.1). The *pHis::PLC* that expresses recombinant PLC were received from Dr. Hitoshi Yagisawa. All expression vectors were in frame with the histidine tag from the plasmid. pLLMP1 plasmid was constructed by cloning rDNA promoter (-265 to +163) from a PCR of the genomic DNA of *B. oleracea* into pGEM. The oligos used for the PCR of rDNA (fwd 5′-TCGGTAC CGAGTTTAGGATGTCAAGT-3′ rev TAGGATCCGGAAAAGTCGCC GGAAAAG-3′) (Chen and Pikaard, 1997). pUC18 was from Thermo Fisher Scientific.

### Recombinant Protein Expression and Purification

Expression vectors were transformed in *Escherichia coli* BL21 (DE3) pLysE from Invitrogen and allowed to grow to an OD of 0.5 at 600 nm. 1 mM IPTG was added after and incubated at 25°C for 3 h. Followed by 10 min centrifugation at 4000 ×*g*, suspension was carried out in a denaturing buffer (20 mM Tris HCl, pH 7.9, 8M Urea, 0.1 M NaH_2_PO_4_, 0.5 M KCl, 20 mM imidazol) and sonicated three times. The re-suspended lysate was centrifuged at 4000 ×*g* for 10 min to remove cell debris and the supernatant was allowed to binding 0.1 ml of Ni^2+^- nitrilotric acetic acid resin for 1 h. The column was wash with 5 ml of the denaturing buffer. Finally 0.3 ml of elution where recovered in a denaturing buffer containing 250 mM Imidazole.

### Nuclear Extract and Histone Purification

*Brassica oleracea* nuclear extraction was carried out as described by Gustavsson et al. (1991). Briefly we used 60 g of fresh weight for the maceration in liquid nitrogen and suspended at 4°C with an extraction buffer 50 mM Tris-Cl pH 8.0, 3 mM EDTA, 2 mM EGTA and 0.2% NP 40. Debris was removed and the extract collected. Centrifugation of the extract was carried out and the nuclei were responded in a hypotonic buffer for 30 min at 4°C followed by addition of an extraction buffer 10 m M Tris-Cl pH 8.0, 1.5 M NaCl, 0.05% NP40 to obtain the NE after centrifugation at 6500 *g* for 10 min. The extraction of histones from *B. oleracea* was carried out from the left over nuclear pellet and high salt extraction buffer 10 m M Tris-Cl pH 8.0, 2.5 M NaCl, 0.05% NP40 was added for 30 min under rotation at 4°C. Centrifugation at 16000 *g* for 10 min. was carried out and the remaining extract contain a large amount of histones.

### Western Blot Analysis

Proteins were separated on a 15% SDS-PAGE and transferred to nitrocellulose membrane (Pall Corporation, USA). After 1h of blocking with 5% non-fat milk in TBST (TBS, 0.1%Tween-20), the membrane was incubated with either anti-H2AQ105 or anti fibrillarin as mention in the legends in TBST with 5% milk over night at 4°C then washed with TBST. Immunoreactive bands were detected with anti-rabbit antibodies conjugated with HRP followed by AlkPhos direct labeling reagents (Amersham).

### Immunofluorescence

The plant tissue was fixed in tubes containing FAA with aspiration for 24 h. They were dehydrated through an ethyl alcohol series and embedded in paraffin (melting point 54–56°C) with a graded series of tertiary butyl alcohol. The paraffin blocks were sectioned serially at 5 μm thickness using a microtome. The deparaffinization was carried out with four washes with Histology grade Xylene for 2 min and by removal of xylene with absolute ethanol. Seventy percent ethanol followed by water for 1 min each. *B. oleracea* inflorescence and surrounding tissue were permeabilized with 0.1% Triton X-100 in PBS for 15 min, respectively. After washes with PBST they were either incubated with anti-H2AQ105me or anti-fibrillarin. Secondary antibodies donkey anti-rabbit IgG conjugated with Alexa 488 (Invitrogen), goat anti-rabbit IgG conjugated with Alexa 647 (Invitrogen). After being washed for 30 min with PBST cells were mounted with moviol (DAPI-DABCO). Images were taken in confocal microscope (Leica TCS SP5 AOBS TANDEM) and a laser-scanning microscope FV100 Olympus with 60X (NA 1.4) oil immersion objective lens. U2OS were treted as published previously (Sobol et al., 2013).

### Gel Mobility Shift Assays

Gel mobility shift assays were carried as previously publish (Castano et al., 1997), with minor modifications. End-labeled rDNA promoter was incubated for 30 min with 10 ng of purified protein at room temperature using the binding reaction contained 5 ng of probe (5000 c.p.m./ng), 25 mM HEPES (pH 7.4), 80 mM NaCl, 10% glycerol, 0.5 mM PMSF and 1 mM leupeptin in a final volume of 20 μl. The misture was separated in a native 6% PAGE at 4°C followed by autoradiography.

### Methylation

For assays on purified histones, 0.2 μg of Atfib2 was assayed on 1 μg of purified histones in the presence of 100 μM SAM (H^3^) in ½ TBS and 1 mM DTT for 30 min at 30°C. Half of the reaction was loaded on SDS–polyacrylamide gel electrophoresis for Coomassie staining and 20% of the reaction for western blotting or for scintillation counting.

### Farwestern

Purified histones were separated on an 15% SDS PAGE and transfer to a PVDF membrane, Membrane was blocked with PBST with 5% of non-fat milk (PBS. 0.1% tween-20) for 1 h at room temperature, then washed three times with PBST. After blocking the membrane was incubated with AtFib2 (0.5 μg) as bait in protein binding buffer (20 mM Tris pH 7.6, 100 mM NaCl, 0.5 mM EDTA, 10% glycerol, 0.1% tween-20, 2% non-fat milk, 1 mM DTT) at 25^a^C for 4 h, then washed three times with PBST and incubated with anti-Fibrillarin (for 12 h at 4^a^C). Immunoreactive bands were detected with anti-rabbit antibodies conjugated with HRP followed by AlkPhos direct labeling reagents (Amersham). Striping of the membrane was incubated at 50°C for 45 min under agitation in a buffer (50 mM Tris HCL pH 6.8, 2% SDS and ß-mercaptoethanol(8ml/l)) followed by rinsing the membrane with water.

### Transcription Pull-down *In Vitro*

Methodology published in Castano et al. (2000). Brief explained a reaction mixture containing either NEs or purified transcription factors were mix with 100 ng of rRNA promoter in the presence of 0.5 mM NTP, 5 mM MgCl, 5 mM DTT, in 20 mM HEPES KOH pH 8.4, in 20 ul reaction volume. In order to assay if H2A methylation was bound during the transcription, rDNA promoter region was bound to magnetic beads (Dynabeads MyOne Streptavidin C1, 650.01, Invitrogen). The promoter was obtained by PCR from the plasmid containing the RNA pol I promoter sequence from *Brassica oleracea*. The oligos used were a 5′-biotin labeled oligo TCGGTACCGAGTTT AGGATGTCAAGT-3′ (promoter region from -265 to -248) and a reverser oligo 5′-TAGGATCCGGAAAAGTCGCCGGAAAAG-3′ from +142 to +163 (published by Chen and Pikaard,1997), Control oligos 5′-biotin labeled pUC18 CCC AGTCACGACGTTGTAA and a reverse CGCAACGCAATTAATGTGAG were purchase from Sigma–Aldrich. Before adding the NEs the bound sequences were blocked with 5% BSA for 1 h at 4°C. The beads were then incubated with NE in a transcription buffer without nucleotides for 1 h, after incubation the beads were washed six times with a buffer containing 20 mM Tris pH 7.9, 100 mM KCl, 0.1 mg/ml BSA, 10% Glycerol, 0.2 mM EDTA pH 8.0. The full amounts of beads were loaded into a PAGE for western blot analysis. PVDF membranes were soaked in Ponceau S stain [0.1% (w/v) Ponceau S in 5% (v/v) acetic acid] to verify protein transfer.

## Results

**FIGURE 1:**
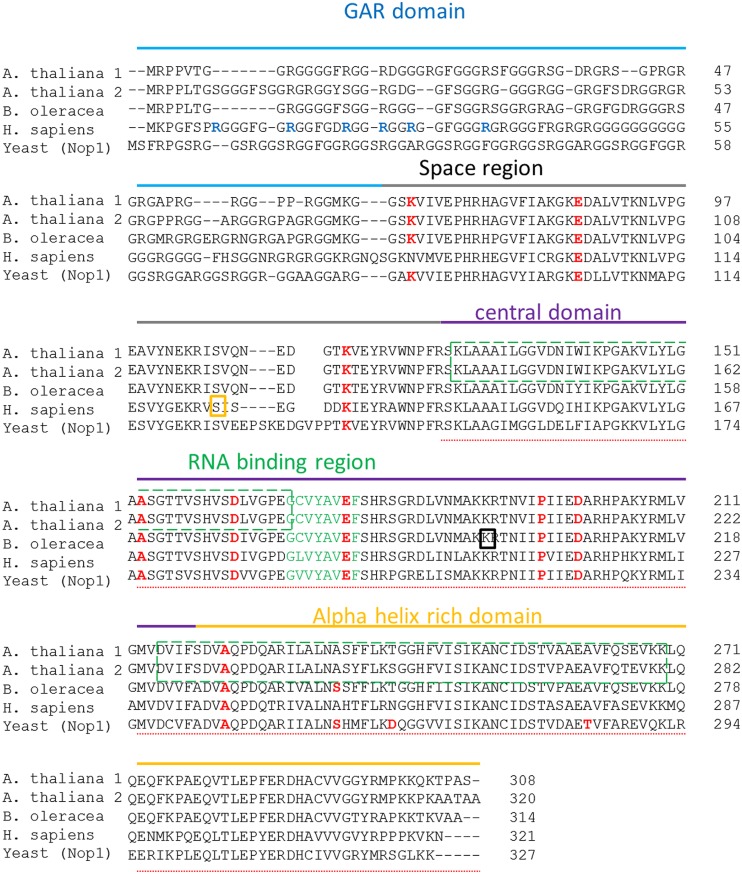
**Fibrillarin sequence comparison relationships of taxa.** The analysis included the sequences from both *Arabidopsis thaliana* fibrillarin (AtFib1 NP_568772.3 and AtFib2 NP_567724.1), fibrillarin sequence from *Brassica oleracea* (BoFib Bol039546), fibrillarin sequence from Homo sapiens (HsFib CAA39935.1) and the yeast fibrillarin Nop1 CAA98572.1. All the domains are label in different colors Gar domain in blue, space region in gray, central domain in purple and the alpha rich domain in orange. Arginines known to be methylated are marked in a red circle. Key amino acids that were mutated in Nop1 are marked in red. The phosphorylated serine is marked in a yellow square and the acetylated lysine in a black square. The doted underline sequence marks the methyl transferase domain. The slash boxes in green indicate the RNA binding domains in *Arabidopsis thaliana* fibrillarins. Green label amino acids indicate the define RNA binding region.

Fibrillarin sequence can be divided into four regions: The GAR domain, Space region, the Central domain with the RNA binding region and the Alpha helix rich domain. The GAR domain is typically the least conserved and contains a non-structural motif that is methylated in human cells. **Figure 1** shows the sequence alignment and domain position for fibrillarins. The comparison between human, yeast, *A. thaliana* and *B. oleracea* reveal that the GAR domain contains the lowest degree of conservation with 31.82% of similarity between AtFib1 and Nop1 as the lowest and with 49.32% of similarity between AtFib2 and BoFib as the highest. The RNA binding domain is well conserved in all species with 72.04% of similarity between AtFib1 and Nop1 as the lowest and with 98.92% of similarity between AtFib1 and AtFib2 as the highest. The alpha helix rich domain differs by 63.44% of similarity between HsFib and Nop1 as the lowest and with 90.72% of similarity between AtFib1 and AtFib2 as the highest, and is known to interact with other proteins in mammalian cells like SMN (Pellizzoni et al., 2001). The red marked amino acids indicate the sites for mutations that allowed Nop1 to be a temperature sensitive mutant. These are key amino acids in Nop1 and are essential for yeast viability at 37°C. We find that for the most part are well conserved, with two alterations between *B. oleracea* and Nop1 located at the N terminus. The two green slash boxes highlight the sequences defined by Rakitina et al. (2011) to be responsible for RNA binding in *Arabidopsis*, while the green letters define the human RNA binding domain. The bold blue label arginine amino acids in the sequence are known to be methylated in human cells. The yellow boxed serine is known to be phosphorylated and the black boxed lysine to be acetylated in human fibrillarin. Although the exact function of all the modifications has still to be defined in any species, and may reflect the high versatility of this protein in different complexes that may occur in the cells (Rodriguez-Corona et al., 2015).

**FIGURE 2:**
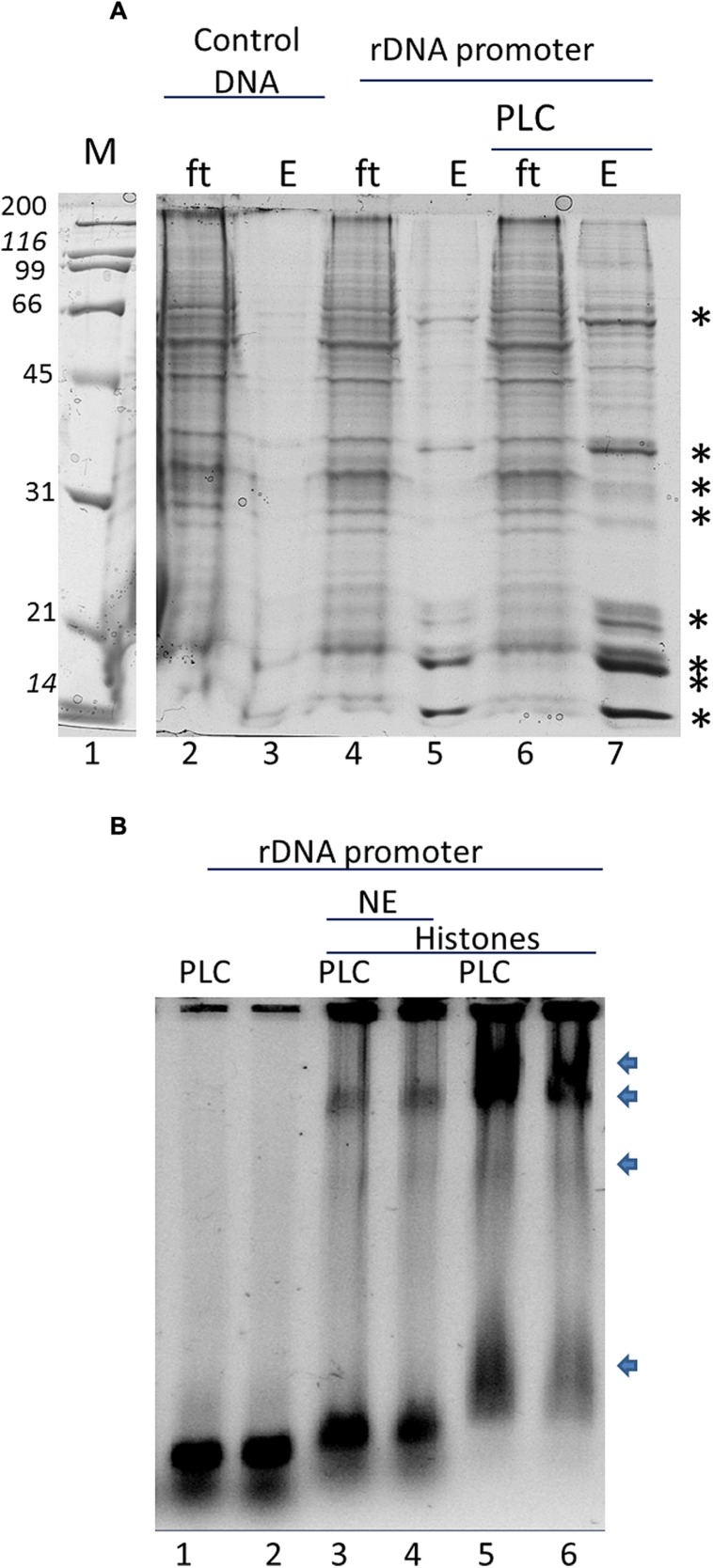
***In vitro* interaction of the rRNA promoter and Histones in *B. oleracea*.** **(A)** rRNA promoter pulldowns of *B. oleracea* nuclear extract. Specific bands label with an ^∗^ represent selective binding proteins to the rDNA promoter. Larger amount was obtained upon PLC treatment to the extract prior to promoter pulldown. **(B)** GMSA with the rDNA promoter. NE was used in combination with purified histones with PLC pretreatment or without the arrows indicates protein-DNA complexes

In order to assay the effect of phospholipids in specific histone binding to the rDNA *in vitro* we tested the NE and purified histones from *B. oleracea* on rDNA promoter binding. We used magnetic beads with the rDNA promoter region as bait. **Figure 2A** shows a typical pull-down experiment, the asterisks indicate the proteins that were specifically bound to the promoter. The amount of these bound proteins increased with the pre-incubation of PLC to the NE. PLC acts on PI4,5P2 which is a known phosphoinositide that binds several nuclear proteins including fibrillarin and histones (Yu et al., 1998; McLaughlin et al., 2002; Yildirim et al., 2013). Since several of the proteins bound to the promoter had a similar profile to that of histones, we further tested that PLC treatment would affect the interaction of histones to the promoter. We carried out a GMSA (**Figure 2B**) with purified histones from *B. Oleracea* and used them to bind the rDNA promoter. Histones bound readily to the promoter and PI4,5P2 degradation by PLC showed an increased histone binding to the promoter. However, the NE reduced significantly the binding of histones to the promoter. This indicates a competition for the interaction between the histones and the ribosomal promoter with NE components that prevent this interaction. PLC treatment under these conditions did not show a significant increase in binding.

**FIGURE 3:**
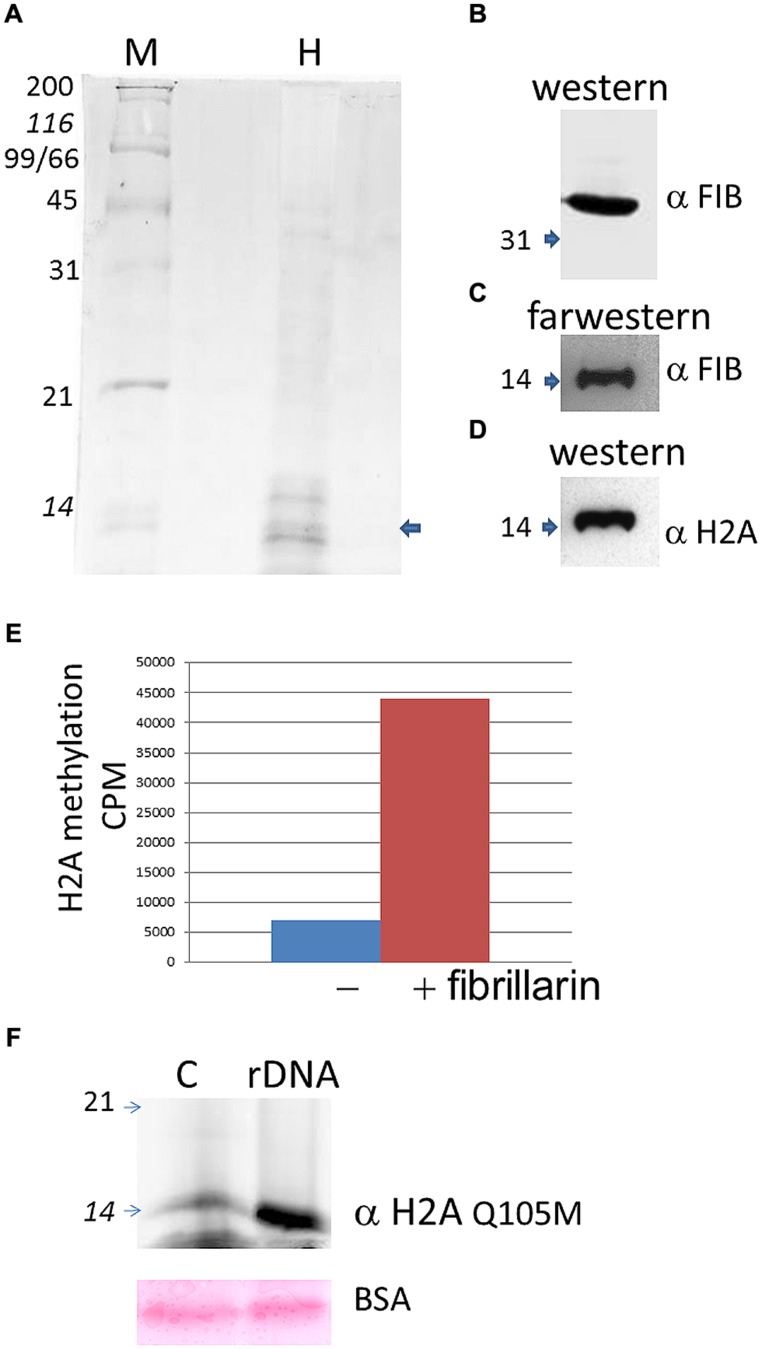
**Histone H2A methylation in *B. oleracea*.** **(A)** Purified histones from *Brassica* as seen in a coomasie stain. M indicate protein weight marker and H the purified histones. The arrow shows the band that is labeled by farwestern in the position of H2A. **(B)**. Western blot of the purified Atfib2. **(C)** Farwestern of the purified histone fraction. Purified Atfib2 was used to screen the histones and find specific interacting partners. **(D)** Western blot of H2A to mark the position of this histone after striping the membrane. **(E)** *In vitro* methylation assay with or without AtFib2 with the *B. oleracea* purified histones. **(F)** Western blot with anti H2A Q105me on histone pulldowns with a control beads (C) or with beads with the rDNA promoter (rDNA). Below is shown the ponceau stain of BSA from the transfer membrane. Numbers indicate the KDA by the marker.

Histone H2A found in active rDNA has been recently shown to be methylated in the nucleolus by fibrillarin in yeast and human cells (Tessarz et al., 2014), therefore we decided to test if plant fibrillarins can also methylate H2A at the rDNA promoter. Purified histones from *B. olearace* were used (**Figure 3A**) and tested for both protein–protein interactions and methylation using AtFib2 (**Figure 3B**) which is 88% identical to BoFib.

To test for protein-protein interactions we used a far-western approach where the transferred histones were used as bait for AtFib2. Western blot of AtFib2 shows the amount used in the assay (**Figure 3B**). The binding of AtFib2 to histone H2A is shown by farwestern (**Figure 3C**). Histone H2A was verified by western blot after stripping (**Figure 3D**). We expected fibrillarin to tightly bind their substrates until the enzymatic reaction could be accomplished plus previously this possible interaction was obtained from a two hybrid system in the interactome data published (Krogan et al., 2006). Krogan et al. (2006) showed H2A among several other proteins that can bind human fibrillarin. After farwestern blot analysis we proceeded to carry out a methylation assay to verify if fibrillarin methylate histones *in vitro* (**Figure 3E**). This was done by mixing tritium radiolabeled SAM, AtFib2 and histones from *B. oleracea*. After the reaction, the histones were separated in a 15% SDS PAGE and stain histones were measured on a scintillation counter showing specific addition of the radiolabeled SAM by the addition of AtFib2.

H2A methylation was further checked by western blot (**Figure 3F**). The aid of anti-H2AQ105me previously used to check H2A methylation by yeast fibrillarin Tessarz et al. (2014) showed successfully that AtFib2 methylate histones H2A from *B. oleracea.* Moreover, AtFib2 methylated H2A while bound to the rDNA promoter. We tested this by allowing the histones from a methylation reaction bind to the rDNA promoter attached to magnetic beads for 1 h. The rDNA promoter bound proteins were resolved on a 15% SDS PAGE and western blot was carried out with anti-H2AQ105me detecting large amounts of methylated H2A as compared with a pUC18 sequence bound to magnetic beads used as control (**Figure 3F**). Since control and rDNA promoter beads were incubated in a buffer containing BSA, the loaded amount was verified by staining the membrane with ponceau and checking that BSA amounts should be equal.

**FIGURE 4:**
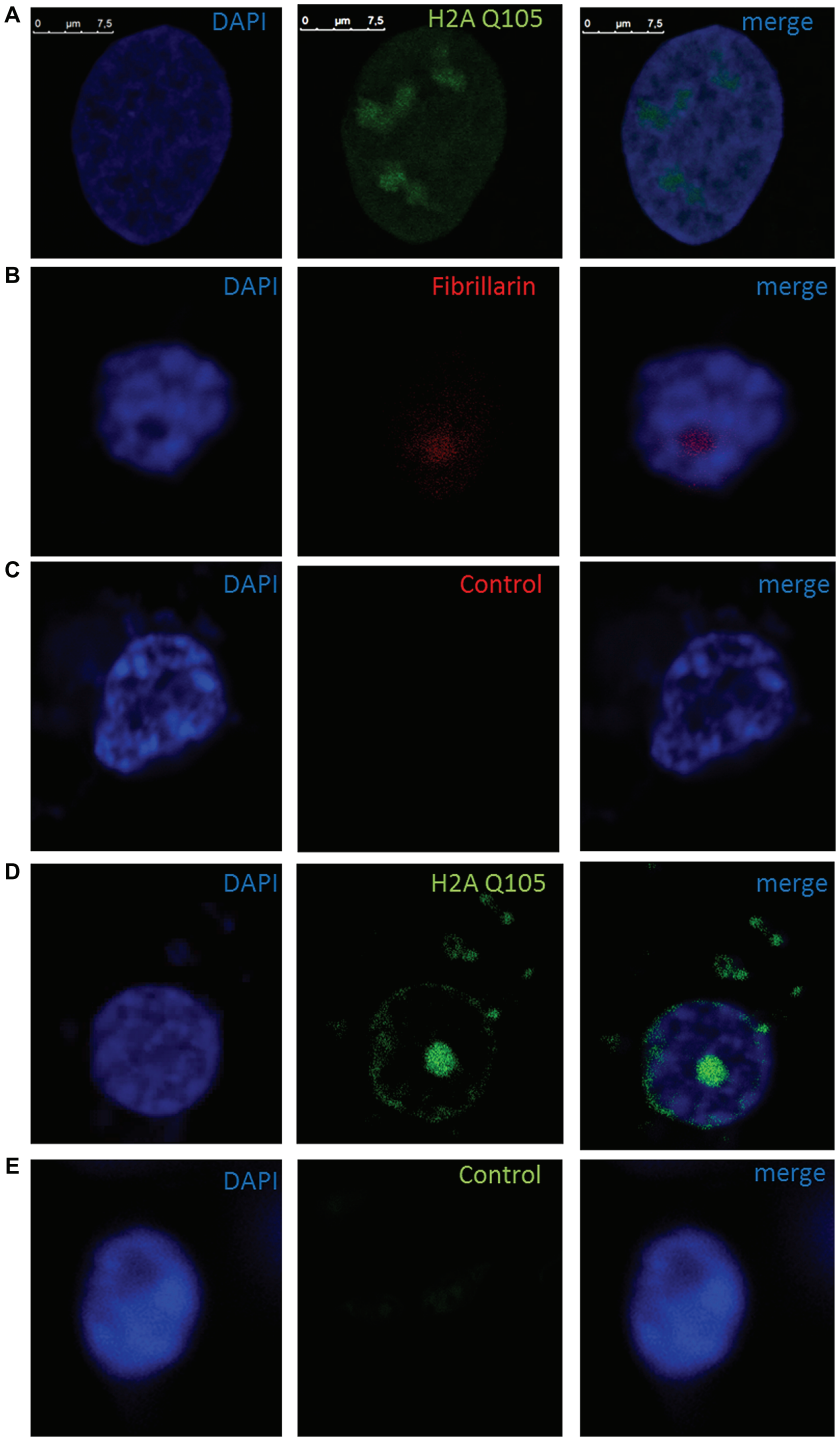
**Immunolocalization of methylated H2A in *B. oleracea*.** All cells where stained with DAPI. **(A)** U2OS cells immunostained with anti H2A Q105me show a specific nucleoli stain. **(B–E)** Nucleus from *B. oleracea* fresh cauliflower floral meristem cells with round nucleus. **(B)** Immunostained with antibodies against fibrillarin. **(C)** Control secondary antibody only couple to alexa 555. **(D)** Imunolocalization of methylated H2A. **(E)** Control secondary antibody only couple to alexa 488.

**FIGURE 5:**
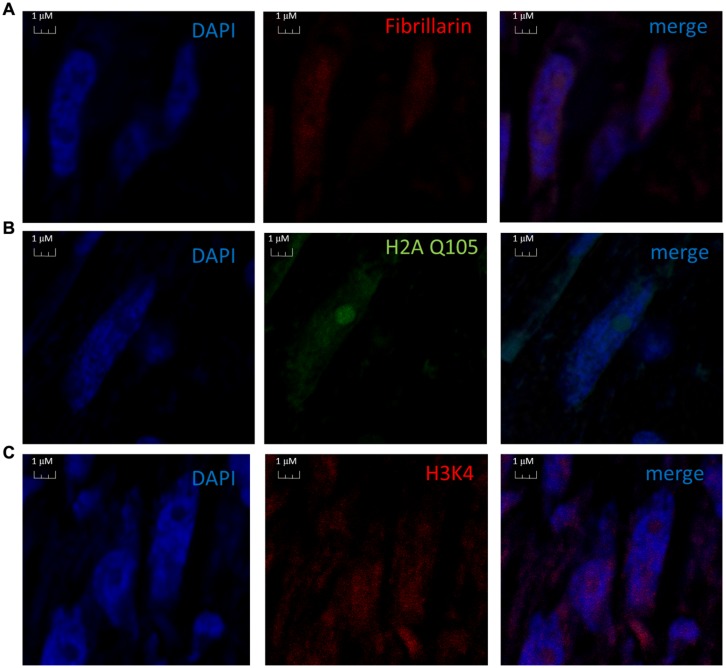
**Nuclear immunolocalization of *B. oleracea* vascular cells.** All vascular cells were stained with DAPI. **(A)** Immunolocalization of Fibrillarin. **(B)** Immunolocalization of methylated H2A showing a similar pattern as fibrillarin immunostain. **(C)** Histone H3 dimethylated in lysine 4 was used as control for nuclear staining a different nuclear pattern from H2A Q105me pattern is observed.

We proceeded with the *in vivo* localization of methylated H2A by immunolocalization in cells of *B. oleracea*. The immunolocalization pattern of anti-H2AQ105me and fibrillarin was compared between human U2OS cells and *B. oleracea* cells. Both plant and human cell lines showed a primary stain of fibrillarin and methylated histone H2A in the nucleoli. Human U2OS cells were used as a control since the immunolocalized pattern for H2A Q105me had already been published (Tessarz et al., 2014). Here we show a higher magnification the staining of anti-H2AQ105me in human cells. As can be seen the stain at the nucleolus is not homogenous and there is a weak diffuse nuclear stain (**Figure 4A**). Floral meristem *B. oleracea* cells showed fibrillarin stain located in the nucleoulus (**Figure 4B**). The secondary antibody did not stain the cells and was used to set the intensities of the signals (**Figure 4C**). The staining with anti-H2AQ105me shows an additional stain on the periphery of the nucleus and additional stain outside the nucleus (**Figure 4D**). This pattern of stain was reproducible in three independent experiments and in all the fresh cauliflower floral meristem buds that have a round nucleus. The staining was specific to anti-H2AQ105me as addition of just secondary antibody did not stain the cells (**Figure 4E**). The additional stain of the anti H2AQ105me outside the nucleus also shows exactly in the same position a weak DAPI stain at the extra nuclear regions, we were surprise by this extra nuclear DNA, but it is been consistent in tree independent experiments with different reagents. This extranuclear DNA could be either an aggregation of organelles like mitochondria from the meristematic cells as previously shown by Kuroiwa et al. (1992). We also checked the pattern in specialized cells. The vascular inflorescence cells showed a different stain as seen in **Figure 5**. Due to the type of tissue, these cells are elongated in order for them to carry out their function. The nucleus is also elongated and thinner than in meristem cells. Here the fibrillarin stain was not only localized to the nucleolus but showed a diffuse stain in most of the nucleus (**Figure 5A**). This is a typical localization of fibrillarin in cells that are under stress (Mironova et al., 2014). As well as in cells that overexpress fibrillarin. Specialized transport cells in plants may reflect this pattern for unknown functional roles at this time. None of these specialized cells showed additional extrachromosomal staining as compared with all of the meristem cells that had a weak extranuclear DAPI stain. The Anti-H2A (Q105M) staining showed a similar pattern to that of fibrillarin. However, these cells had no perinuclear staining or additional extra nuclear stain (**Figure 5B**). These are the first results that show nucleolar methylated histone H2A in plants and may involve a conserved epigenetic rDNA transcriptional mechanism for all eukaryotic cells nucleoli. The immunolocalization of Dimethylated lysine 4 in histone 3 in these cells shows an overall nuclear pattern, with no selectivity for the nucleolus as compared with methylated H2A (**Figure 5C**). Furthermore, the methylation of histone H2A in specialized cells can be involved in other epigenetic mechanism that can be specific to plants outside the nucleoli as shown by the immunolocalization pattern of H2A.

## Discussion

Fibrillarin sequence in all eukaryotic cells differs from Archaea organisms by addition of the GAR sequence (Rodriguez-Corona et al., 2015); this highly methylated region is responsible for nucleolar localization and protein–protein interaction and is the less conserved sequence in all fibrillarins (Snaar et al., 2000). The lack of conservation in the GAR domain can indicate that only methylated arginine charges are involve for these activities. Although up to date, there is no biochemical data that provides clear function besides the nucleolar localization (Rodriguez-Corona et al., 2015). The central domain, the RNA binding region and the Alpha helix rich domain form the methyl transferase region that allow fibrillarin to methylate rRNA and histones. Tessarz et al. (2014) showed recently that yeast and human fibrillarin can methylate histone H2A and the previously thermo-sensitive yeast fibrillarin (Nop1) mutant (Tollervey et al., 1993) showed a reduction of methyl transferase activity of H2A from Nop1 at the non-permissive temperature after 3 h (Tessarz et al., 2014). Thus showing that Nop1 is responsible for this methylation and the methylation is under constant evaluation by the cell. Probably this is part of the mechanism that helps the cell define the number of ribosomal promoter regions that to be active. Interestingly the key mutated amino acids in the alpha helix in yeast are not well conserved in plants as seen in **Figure 1**. This may reflect the difficulty of some fibrillarins to recapitulate fully all the functions of fibrillarin in a yeast complementary assay (Jansen et al., 1991; Pih et al., 2000). The promoter of the rDNA from *B. oleracea* was reported by Chen and Pikaard (1997) and has been used previously *in vitro* transcription assays. We used the same assay as bait for nuclear proteins in particular histones and fibrillarin. Since PI4,5P2 is known to interact with fibrillarin and histones we tested if the degradation of PI4,5P2 by the recombinant PLC added into the assay would alter the amount of nuclear proteins that bind the promoter. The results correlates with the studies on histone H1 and H3 interaction with PI4,5P2 where it was suggested that this lipid may promote the formation of less accessible interaction of RNA pol II to the promoter due to higher binding of the histones (Yu et al., 1998). PI4,5P2 is well known phosphoinositide in the signal transduction mechanism in the cell membrane (McLaughlin et al., 2002; Lemmon, 2008; Boss and Im, 2012), where it is digested by PLC into PIP3 and DAG. However, the nuclear form of this lipid has only come into play during the last decade (Osborne et al., 2001; Yildirim et al., 2013). PI4,5P2 is known to bind histone H1, H3 as well as fibrillarin, StarPAP, UBF etc. (Yu et al., 1998; Jiang et al., 2006; Mellman et al., 2008; Yildirim et al., 2013) and localized in transcriptionally active ribosomal promoters in human cells. Up to date it is not clear what is the mechanism by which PI4,5P2 is affecting transcription and it’s interesting that its removal increases binding of several proteins to the rDNA promoter as seen in **Figure 2**. H1 was reported to increase its binding activity as a result of PI4,5P2 loss (Yu et al., 1998). However, there is no studies yet done on chromatin structure alteration by phosphoinositides.

The *in vitro* methylation of *B. oleracea* histones by AtFib2 is similar to the results obtained recently by Tessarz et al. (2014) with purified Nop1. This epigenetic mechanism involves fibrillarin marking histone H2A on active ribosomal promoters. Our pulldown experiments with the rDNA promoter show a preferential binding of methylated H2A as compared to a control sequence. As previously publish that fibrillarin and histone bind well to the human rDNA promoter (Yildirim et al., 2013). On a recent model (Leonhardt and Hake, 2014). Fibrillarin interacts with RNA pol I and such interaction represses FACT complex action on chromatin remodeling. This model is interesting considering that fibrillarin in plants has been shown to be part of the mediator for RNA pol II transcription, as up to date there is a missing functional data to explain the function of fibrillarin on the mediator. It may help in the process of chromatin remodeling in other parts outside the nucleolus. It was observed on the immunolocalization of both fibrillarin and methylated histone H2A in the *B. oleracea* nucleus. There is a clear label outside the nucleolus in plant cells that is not seen in human U2OS cells. This may indicate plant fibrillarin role with RNA pol II, however more experiments are required to test this hypothesis. Fibrillarin is primarily located in the nucleoli, in particular in the DFC and FC regions. However, in these regions, several processes take place, the transcription initiation, elongation and first stages of rRNA processing take place in this region and may involve different functions of fibrillarin, which is well known to methylate rRNA for further processing. Methylation of H2A may help discriminate between active and inactive rDNA and its nucleoli organization. There is evidence that core histone H3 is also located in mitochondria in *B. oleracea*, however, this is not recognize by highly specific antibodies for the N terminal tail region of H3. One possibility is that the N terminal region of H3 is modified and is not detected with these antibodies (Iwasaki et al., 2013). A similar scenario could explain the methylated H2A signal in the extra nuclear stain in the fresh cauliflower floral meristem buds. Since *B. oleracea* meristem cells are the most exposed cells it would follow that it may also have this additional function. Furthermore other explanation may involve ribonucleoproteins known to interact with fibrillarin that can form U bodies structures found in the cytoplasm (Liu and Gall, 2007) although it is unclear why methylation of H2A would be required for this outside the nucleus. Although it is known those histones H2A/H2B have antimicrobial action in particular cells that are closer to the surface as publish (Stekhoven et al., 2004). The absence of this signal in vascular inflorescence cells can be due to a reduction in the number of mitochondria for this cell type or lack of U bodies. Plant viruses that interact with fibrillarin may take advantage of the broad distribution of this protein in this transport cell. The spread of the virus through the plant aided by fibrillarin has been published (Kim et al., 2007) and the diffusion of fibrillarin in vascular cells may help viruses tag alone for distribution through the phloem. The diffusion of the methylated H2A in transport cells correlates well with the diffusion pattern of fibrillarin. However, it is early to define the role of this epigenetic marker and its functional significance in this type of cells. Tessarz et al. (2014) had shown a particular interaction with FACT and it is known that in many cell types FACT facilitates the remodeling of RNA pol II promoter more than RNA pol I promoters. Recently it was shown that FACT–Histone interactions identifies a role of Pob3 C-terminus in H2A–H2B binding (Hoffmann and Neumann, 2015). So it is possible that methylation of H2A in specialized cells may reflect this interaction as suggested by Hoffmann and Neumann that FACT interactions are altered by histone posttranslational modification.

## Acknowledgment

We would like to thank to Angela Ku and Wilma A. Gonzalez for their technical help.

## Conflict of Interest Statement

The authors declare that the research was conducted in the absence of any commercial or financial relationships that could be construed as a potential conflict of interest.

## ABBREVIATIONS

aFib: archaea fibrillarin
AtFib1: *Arabidopsis thaliana* fibrillarin 1
AtFib2: *Arabidopsis thaliana* fibrillarin 2
BoFib: *Brassica oleracea* fibrillarin
DABCO: 1,4-Diazabicyclo(2.2.2)octane
DAG: diacylglycerol
DAPI: 4′,6-diamidino-2-phenylindole
DFC: dense fibrillar component
DTT: dithiothreitol
FAA: formalin – acetic acid – alcohol
FACT: facilitator of chromatin transcription
FC: fibrillar center
GAR: arginine glycine rich domain
GC: granular component
GMSA: gel mobility shift assays
HRP: horseradish peroxidase
HsFib: homo sapiens fibrillarin
IPTG: Isopropylthiogalactoside
IRES: internal ribosome entry site
NCBI: national center for biotechnology information
NE: nuclear extract
Nop1: nucleolar protein 1
Nop56: nucleolar protein 56
Nop58: nucleolar protein 58
PBS: phosphate-buffered saline
PBST: Phosphate-buffered saline tween
PI4,5P2: phosphatidylinositol 4,5-bisphosphate
PIP3: phosphatidylinositol 3,4,5 trisphosphate
PLC: phospholipase C
PVDF: polyvinylidene difluoride membrane
rDNA: ribosomal DNA
rRNA: ribosomal RNA
SAM: *S*-adenosyl methionine
SMN: survival of motor neuron
snoRNA: small nucleolar RNA
snoRNP: small nucleolar ribonucleoprotein
TBS: Tris-buffered saline
TBST: Tris-buffered saline tween
U2OS: human osteosarcoma cell line
UBF: upstream binding factor
